# Trend of *Entamoeba histolytica *infestation in Kolkata

**DOI:** 10.1186/1757-4749-2-12

**Published:** 2010-10-06

**Authors:** Avik K Mukherjee, Kaushik Das, Mihir K Bhattacharya, Tomoyoshi Nozaki, Sandipan Ganguly

**Affiliations:** 1 Department of Parasitology, National Institute of Cholera and Enteric Diseases, Indian Council of Medical Research, P33 CIT Road, Scheme XM, Kolkata - 700010, India; 2 Department of Clinical Medicine, National Institute of Cholera and Enteric Diseases, Indian Council of Medical Research, P33 CIT Road, Scheme XM, Kolkata - 700010, India; 3 Division of Parasitology, National Institute of Infectious Diseases, Shinjuku, Tokyo, Japan

## Abstract

**Background:**

*Entamoeba histolytica* infection is found almost all over the world and is highly endemic and a major cause of parasitic diarrhoea particularly in the developing countries.

**Methods:**

A systemic surveillance was set up at the Infectious Disease hospital, Kolkata, India between November 2007 and October 2009 for understanding the trend of *E. histolytica* infection in Kolkata. Fecal samples were collected from diarrhoeal patients attending the hospital, under the surveillance system and processed for detection of *E. histolytica*.

**Results:**

During the last two years about 2500 diarrhoeal samples were collected and screened for *E. histolytica*. About 3.6% were positive for *E. histolytica*. As compared to the earlier years, *E. histolytica* infection was observed to be less amongst patients screened during the last two years. No seasonality was observed in Kolkata although in the neighboring tropical country Bangladesh, a typical seasonality of *E. histolytica* infection was noticed.

**Conclusion:**

The study indicates that the detection rate of *E. histolytica* infection amongst diarrhoeal patients in Kolkata is decreasing during the last two years than that of Bangladesh.

## Background

Amoebiasis caused by infection with *E. histolytica *occurs almost all over the world and is highly endemic especially in the developing countries. It is one of the major causes of dysentery/diarrhoea in Kolkata, India. According to our previous study (unpublished), detection of *Entamoeba histolytica *showed a marked seasonality, i.e. high peak during post-monsoon and post-winter seasons. According to the reports from other tropical countries, especially in Bangladesh, which is geographically closest to Kolkata, there is a typical pattern of detection of *E. histolytic w*here *E. histolytica *usually shows its highest peaks in the wet season and gradually decreases with the arrival of dry season [1,2]. In a study in Bangladesh, it was shown that wet environment is not the only factor that affects the detection curve of *E. histolytica*, but anti-Carbohydrate Recognition Domain IgA level in the gut is another determining factor for its occurrence in a closed population [3]. However, even in that case *E. histolytica *detection followed a particular seasonality and trend. However in Kolkata the present scenario is different from other tropical regions.

## Study design and results

**Figure 1:**
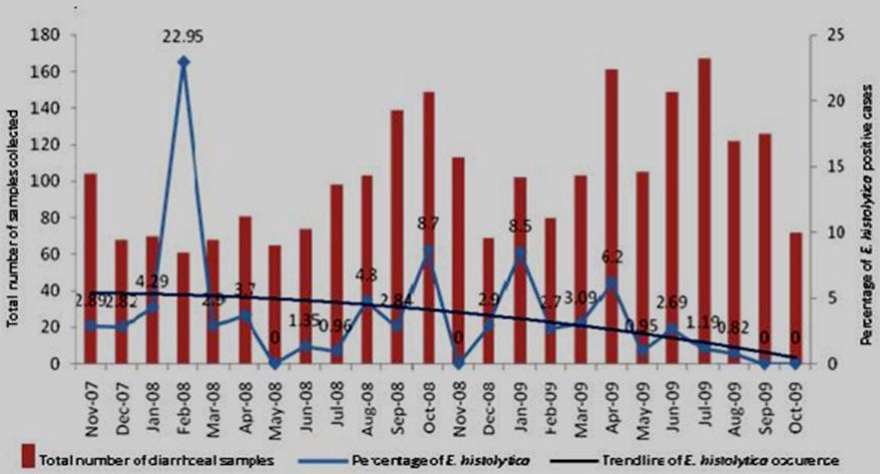
**Month wise distribution of samples collected throughout the study period along with percentage of *E. histolytica *positive among them**.

For the last few years, we have been engaged with the surveillance study to determine the detection rate of common enteric parasites in Kolkata. During the last two years, about 2500 diarrhoeal stool samples were screened from hospitalized patients through a systematic sampling procedure as described previously[1] in collaboration with Infectious Diseases Hospital, Kolkata. In this surveillance, every fifth patient attending the Infectious diseases hospital from different parts of Kolkata with diarrhoea in randomly selected two days a week were included in the study. Fecal samples from these patients were collected and sent to the laboratory within half an hour of collection for detection of *E. histolytica *by microscopic examination using iodine wet mount method; PCR and antigen capture ELISA. The whole study was carried out from the month of November 2007 to October 2009 to include at least two seasonal cycles for better understanding of the seasonality. Although one of the major diarrhoea causing pathogen in Kolkata is *Vibrio cholerae *[4] among all etiologies, *E. histolytica *(3.6%) is one of the major diarrheagenic parasites among the parasitic etiologies of diarrhoea. It caused some sporadic diarrhea throughout the study period without any particular seasonality which is uncommon for a tropical area like Kolkata. Overall a decreasing trend of *E. histolytica *infection was also seen (Figure 1). Reasons behind this different nature of infection might be due to physical and environmental factors which could influence *E. histolytica *detection rate, such as choice of common antimicrobial drug, temperature, detection procedure of *E. histolytica *etc. But none of these changed strikingly in last two years that could affect *E. histolytica *detection rate. Indiscriminate use of anti-parasitic drugs like metronidazole and tinidazole could be attributed to the decreasing trend of detection of *E. histolytica *in Kolkata.

## Conclusion

The present study demonstrates that there is no particular seasonality of occurrence of *E. histolytica *infection which is not typical of a tropical area like Kolkata. A different pattern which is almost unique in comparison to other tropical countries has been observed. Contribution of environmental and host factors as well as parasite genotypes are very important for the outcome of infection [2,5]. Although no physical or environmental factor behind this changing pattern of *E. histolytica *infestation in Kolkata has yet been reported, but it is certain that *E. histolytica *is showing a slow but obvious change in its seasonality and this might be a signal for a transition period of changing nature of infestation by this parasite in this part of the world. It is worth mentioning that impact of climate change might lead to such changes, although no such studies are available.

## References

[B1] Mukherjee AK Chowdhury P Bhattacharya MK Ghosh M Rajendran K Ganguly S Hospital-based surveillance of enteric parasites in Kolkata BMC Research Notes 2009 2 110 10.1186/1756-0500-2-110 19545355 PMC2706841

[B2] Ravdin JI Amebiasis; Series on Tropical Medicine Science and Practice 2000 2 Imperial College Press 55 10.1023/A:1017235624216

[B3] Haque R Mondal D Duggal P Kabir M Roy S Farr BM Sack RB Petri WA Jr *Entamoeba histolytica *Infection in Children and Protection from Subsequent Amebiasis Infect Immunity 2006 904 909 10.1128/IAI.74.2.904-909.2006 PMC1360358 16428733

[B4] Nair Gopinath Balakrish Ramamurthy Thandavarayan Bhattacharya Mihir Kumar Krishnan Triveni Ganguly Sandipan Saha Dhira Rani Rajendran Krishnan Manna Byomkesh Ghosh Mrinmoy Okamoto Keinosuke Takeda Yoshifumi Emerging trends in the etiology of enteric pathogens as evidenced from an active surveillance of hospitalized diarrhoeal patients in Kolkata, India Gut Pathogen 2010 2 4 10.1186/1757-4749-2-4 PMC2901208 20525383

[B5] Petri WA Jr Mondal D Peterson KM Duggal P Haque R Association of malnutrition with amebiasis Nutr Rev 2009 67 S207 10.1111/j.1753-4887.2009.00242.x 19906225

